# Antibody-Drug Conjugates in Solid Tumor Oncology: An Effectiveness Payday with a Targeted Payload

**DOI:** 10.3390/pharmaceutics15082160

**Published:** 2023-08-19

**Authors:** Aleksei Kondrashov, Surendra Sapkota, Aditya Sharma, Ivy Riano, Razelle Kurzrock, Jacob J. Adashek

**Affiliations:** 1 Department of Internal Medicine, Saint Agnes Hospital, Baltimore, MD 21229, USA; dr.akondrashov@gmail.com (A.K.); surendra.sapkota@ascension.org (S.S.); 2 Department of Internal Medicine, Dartmouth Health, Geisel School of Medicine at Dartmouth, Lebanon, NH 03756, USA; aditya.sharma@hitchcock.org (A.S.); ivy.riano@dartmouth.edu (I.R.); 3 Division of Hematology and Oncology, Dartmouth Cancer Center, Lebanon, NH 03755, USA; 4 WIN Consortium, 94550 Paris, France; rkurzrock@mcw.edu; 5 MCW Cancer Center, Milwaukee, WI 53226, USA; 6 Division of Oncology and Hematology, University of Nebraska, Omaha, NE 68198, USA; 7 Department of Oncology, The Sidney Kimmel Comprehensive Cancer Center, The Johns Hopkins Hospital, Baltimore, MD 21287, USA

**Keywords:** precision oncology, targeted therapy, agnostic targets, antibody–drug conjugates, novel therapeutics, solid tumors

## Abstract

Antibody–drug conjugates (ADCs) are at the forefront of the drug development revolution occurring in oncology. Formed from three main components—an antibody, a linker molecule, and a cytotoxic agent (“payload”), ADCs have the unique ability to deliver cytotoxic agents to cells expressing a specific antigen, a great leap forward from traditional chemotherapeutic approaches that cause widespread effects without specificity. A variety of payloads can be used, including most frequently microtubular inhibitors (auristatins and maytansinoids), as well as topoisomerase inhibitors and alkylating agents. Finally, linkers play a critical role in the ADCs’ effect, as cleavable moieties that serve as linkers impact site-specific activation as well as bystander killing effects, an upshot that is especially important in solid tumors that often express a variety of antigens. While ADCs were initially used in hematologic malignancies, their utility has been demonstrated in multiple solid tumor malignancies, including breast, gastrointestinal, lung, cervical, ovarian, and urothelial cancers. Currently, six ADCs are FDA-approved for the treatment of solid tumors: ado-trastuzumab emtansine and trastuzumab deruxtecan, both anti-HER2; enfortumab-vedotin, targeting nectin-4; sacituzuzmab govitecan, targeting Trop2; tisotumab vedotin, targeting tissue factor; and mirvetuximab soravtansine, targeting folate receptor-alpha. Although they demonstrate utility and tolerable safety profiles, ADCs may become ineffective as tumor cells undergo evolution to avoid expressing the specific antigen being targeted. Furthermore, the current cost of ADCs can be limiting their reach. Here, we review the structure and functions of ADCs, as well as ongoing clinical investigations into novel ADCs and their potential as treatments of solid malignancies.

## 1. Introduction

The development of antibody-drug conjugates (ADCs) has been a major advance in the oncology field. ADCs consist of a monoclonal antibody (mAb) that is covalently linked to a cytotoxic drug via a chemical linker; the cytotoxic agent is known as the “payload”. The concept of ADCs, also known as the “magic bullet” was first proposed by Paul Ehrlich in 1907, yet they were first used in clinical trials only in the 1980s [1]. The intensity of ADC research has been steadily increasing over the past decade [2]. Notably, most ADCs are approved for hematological malignancies, and progress in solid tumor ADCs differs from that in hematological malignancies. The field of ADCs faces significant challenges including the complexity of the drug development, its cost, evolving tumor biology and multiple mechanisms of resistance.

Currently, there are dozens of ADC agents, and their scope of use is increasing. Gemtuzumab ozogamicin (Mylotarg^®^) (CD33-directed antibody drug conjugate linked to the cytotoxic antibiotic calicheamicin) was the first ADC drug approved by the U.S. Food and Drug Administration (FDA). It was initially approved in 2000 for the treatment of adults with CD33-positive acute myeloid leukemia (AML), marking the beginning of the ADC era in targeted therapies; it was, however, withdrawn from the market in 2010 because of failure of a confirmatory trial and safety concerns; it was then re-approved in 2017, using a lower dose fractionated regimen, for treatment of relapsed or refractory (R/R) CD33-positive acute myeloid leukemia (AML) in patients two years of age and older [3]. In regard to solid tumors, in 2013, the FDA approved ado-trastuzumab emtansine (T-DM1) (payload is a microtubule inhibitor) for the treatment of metastatic breast cancer on the basis of its HER-2 targeting [4]. Another first-in-class ADC, trastuzumab deruxtecan (T-Dx) (payload is a topoisomerase I inhibitor), was first approved in 2021; currently, it is approved for HER2-positive breast cancer and, more recently, HER2-low (immunohistochemistry 1+ or 2+) breast cancer, HER2 positive gastric or gastroesophageal cancer, and *HER2*-mutated non-small cell lung cancer (NSCLC) [5,6,7,8].

Our objectives for this review were to provide up-to-date information about FDA-approved ADCs for solid tumors, their history and structure, side effects profiles, available clinical trial data, and prospective agents in development and to outline major barriers for their broader clinical use.

## 2. General ADC Structure

Since the 1970s, there have been innumerable approaches to ADC synthesis and development, for both solid and hematological tumors. Multiple types of drug-conjugates were proposed, such as bispecific ADCs, bi-epitope ADCs, fab-fragment of the antibody (FabDCs), single-chain antibody fragment drug conjugates, nanobody drug conjugates, peptide drug conjugates, radioimmune conjugates, bispecific monoclonal antibodies (mAb), dual payloads, nanoparticles, and others [9].

**Figure 1:**
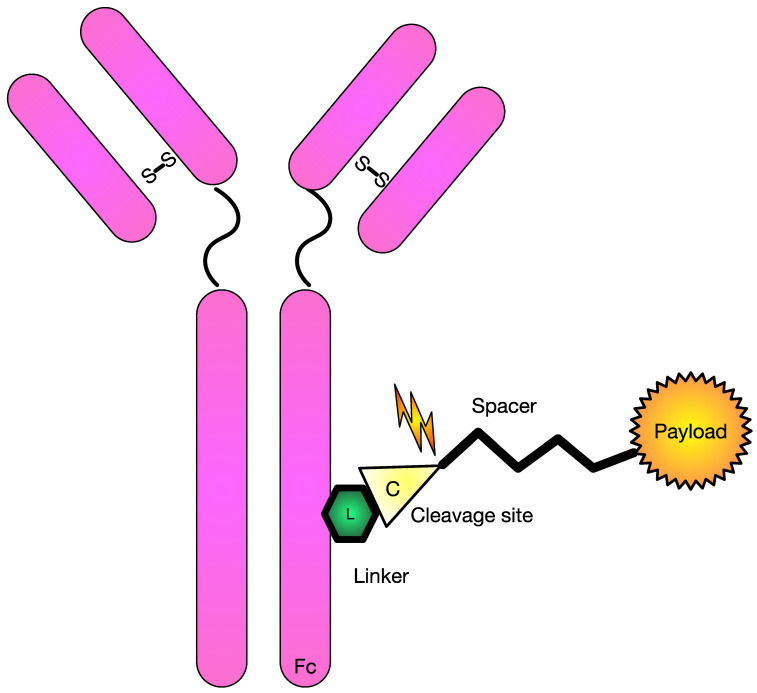
Schematic diagram of commonly used antibody drug-conjugate. Figure Legend: This diagram shows a common monoclonal antibody with the necessary pieces to conjugate it to a cytotoxic agent. In this schematic, there includes a linker piece to a cleavage site that can be cleaved and allow delivery of the payload.

**Figure 2:**
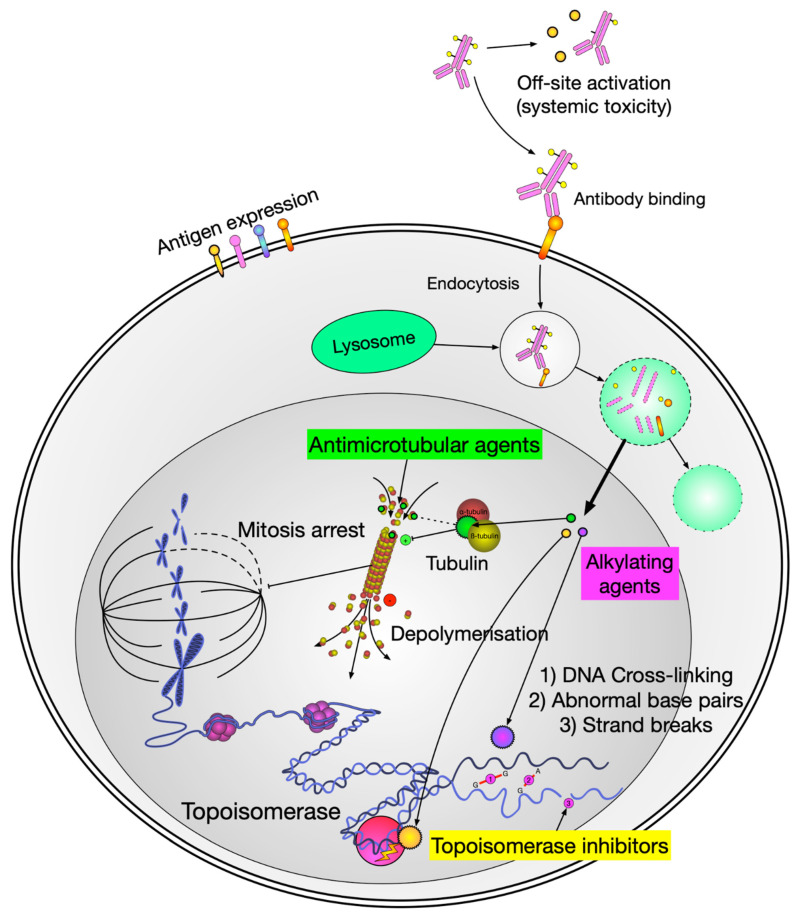
Schematic diagram of payload delivery and its mechanism of action. Figure Legend: When the antibody engages with the antigen of the target cell, the ADC enters the cell and through the intracellular lysosome is cleaved, which then releases the cytotoxic payload to induce cellular death via various mechanisms.

Modern ADCs typically consist of three main components: an antibody, a linker, and a payload (Figure 1). Most ADCs undergo similar mechanisms to release the payload. In general, after intravenous (IV) administration and tissue distribution, ADCs bind target antigens with the antibody moiety and undergo internalization/endocytosis. In the endosomes (if cleavable), the payload is released via protease (such as cathepsin B) cleavage of the linker or by the degradation of the ADC. The activated payload diffuses to cytosol and the nucleus, binds to microtubules or DNA, and triggers cell death (Figure 2). If lipophilic, the activated payload can diffuse into the microenvironment and kill neighbor cells (bystander effect) [10].

**Figure 3:**
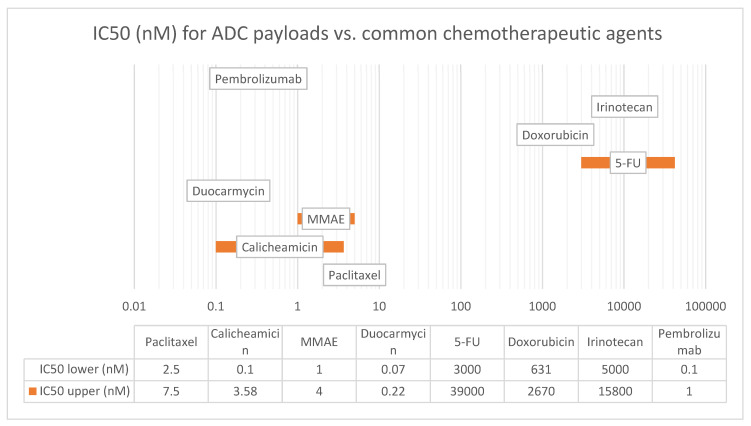
IC50 (nM) for ADC payloads vs. common chemotherapeutic agents. Figure Legend: IC50 is a measure of the potency of a drug and represents the concentration of the drug required to inhibit the growth of 50% of the cells in vitro. The lower the IC50 value, the more potent the drug. The IC50 values listed in this table are representative values and vary depending on the cell line (tumor/tissue type) used and the experimental conditions (drug concentration, exposure time, environment). Abbreviations: 5-FU—5 fluorouracil, MMAE—monomethyl auristatin E.

Depending on linker technology, different proportions of the payload may be systemically released, contributing to systemic toxicity, or acting as traditional chemotherapy. In some cases, the systemic payload release was so significant that concerns were raised if there was any ADC attributed activity at all [11]. Some of these concerns are also based on studies suggesting that less than 1% of the administered ADC reaches the target tumor site [12]. Radiolabeled antibody studies showed that only 0.01% of the injected antibody could be localized in a solid tumor mass 24 h after infusion, irrespective of tumor type or antibody target [13]. The immunoglobulin G1 (IgG1) antibody molecular mass is 146 kDa [14]. Typical payloads, such as monomethyl auristatin E (MMAE), have molecular mass of 0.7 kDa [15]; which, combined with valine–citrulline (vc) protease cleavable linker, provides a mass of 1.3 kDa [16]. Lastly, the quantity of payload molecules (drug-to-antibody) ratio is limited by chemical properties of the payload and linker, and for most of the FDA-approved agents is four (Table 1) [17,18]. MMAE-derived hydrophobic payloads tend to aggregate with DARs > 4 [19]. The payload of four vc-MMAEs would constitute about 3.4% (5.2/151.2 kDal) of IgG1 ADCs molecular mass. Thereby, the payload must be so potent that it would be active in the nano- or even picomolar range (Figure 3 and Supplemental Figure S1) [20].

Some of the recently approved agents, such as Sacituzuzmab govitecan (Trodelvy) and Trastuzumab Deruxtecan (Enhertu) and have a much higher DAR of 7–8. Both Enhertu and Trodelvy are topoisomerase I inhibitor-based ADCs, structurally analogous to plant-derived alkaloid camptothecin [29]. What is new that both conjugates are stable and have high clinical efficacy. It has been hypothesized that the topoisomerase inhibitor payload type [30] or more hydrophobic chemical structure for both payloads of Enhertu and Trodelvy increased their bystander killing, but kept conjugates stable [31].

### 2.1. Payload Types

The cytotoxic payload is an effector molecule of ADCs. The list of payloads is extensive, including various alkylating agents, topoisomerase inhibitors, immune stimulants, RNA polymerase inhibitors, tyrosine kinase inhibitors, protein synthesis inhibitors, proteolysis targeting chimera, and others [17,32,33,34].

Despite the wide range of studied agents, there are only three payload classes in FDA-approved agents: microtubular inhibitors (auristatins (MMAE), maytansinoids (DM1 and DM4)), topoisomerase inhibitors (deruxtecan, SN-38—active form of irinotecan), alkylating agents (duocarmazine). All other agents are still under current investigation or only used in hematological malignancies.

The development of alternative (noncanonical) payloads is a field of active research. One such example is the recombinant immunotoxin composed of an anti-CD22 Ab and a Pseudomonas exotoxin A fragment (Moxetumomab pasudox), which has been approved for the treatment of hairy cell leukemia [35]. ADCs may also carry immunoenhancing molecules such as interleukins or immune checkpoint inhibitors (ICIs), such as PD-1/PD-L1 blockers [36], to increase local concentration and enhance the localized tumor immune response. Another experimental type of payload is Target Protein Degradation (TPD) using degrader–antibody conjugates (DACs) [37]. Although combinations of ADCs and ICIs may exhibit synergistic effects theoretically [38], in the KATE2 trial, addition of atezolizumab to trastuzumab emtansine was not shown to significantly improve progression-free survival and was associated with increased adverse events [39].

#### 2.1.1. Auristatins

MMAE and monomethyl auristatin F (MMAF) are derivatives of dolastatins, a group of peptides extracted from the sea hare mollusk *Dolabella auricularia* in the late 1970s. Auristatins are a family of protein derivatives of dolastatin 10. Auristatins bind tubulin in the same domain as vinca alkaloids (such as vincristine) and cause metaphasic arrest. In vitro activity of dolastatins against certain leukemia cell lines led to their evaluation in several clinical trials for several types of cancer; however, no significant in vivo activity was observed. Twenty years later, in the 1990s, with the aim of improving the potential of dolastatins, MMAE and MMAF were developed with a linker for antibody conjugation. Nowadays, MMAE is the most studied payload (Table 1 and Table 2). However, no MMAF-based ADCs have been approved by the FDA yet [40].

#### 2.1.2. Maytansinoids (DM1 and DM4)

Maytansinoids represent a second class of microtubule inhibitors derived from the naturally occurring maytansine, a benzoansamacrolide that is isolated from the bark of the African shrub *Maytenus ovatus*. Maytansine also binds tubulin at the vinca-binding site. The maytansinoid drugs are 200- to 1000-fold more cytotoxic than other conventional chemotherapeutic agents. Maytansine in its original form yielded a narrow therapeutic window due to associated neurological and gastrointestinal toxicities and appears to be only suitable in ADC setting [41].

#### 2.1.3. Calicheamicin

Calicheamicin is an antibiotic derived from the soil-dwelling bacterium *Micromonospora echinospora calichensis*. It is one of the most potent chemotherapeutic agents ever known. It was first discovered in mid-1980s by a scientist from “Calicheamicin works” by inducing a Bergman cyclization reaction, which results in DNA cleavage and cell death. It is an active payload of Mylotarg^®^ (gemtuzumab ozogamicin) and inotuzumab ozogamicin. Both agents are used in leukemias. Despite its potential, the clinical development of Calicheamicin has been challenging due to the compound’s inherent toxicity and the difficulty in selectively delivering it to cancer cells.

#### 2.1.4. Duocarmycins

Ones of the most promising new payloads in solid tumors are duocarmycins. These are DNA-alkylating agents isolated from *Streptomyces* sp. with a unique chemical structure that covalently binds to the deoxyribose in DNA, leading to DNA damage and apoptosis. They were first isolated from the genus Streptomyces bacteria in 1978 [42]. Duocarmycins are extremely cytotoxic and active at picomolar concentrations, making them good candidates for ADC development. Since their invention, hundreds of molecules have been investigated, however, there have not been any approved duocarmycin-based ADCs yet [43].

### 2.2. Linkers

The linker is a critical component of any ADC. The nature of the linker and spacer determines important features of ADCs, such as hydrophobic or lipophilic properties of payload, nontarget payload delivery, systemic toxicity, and most importantly, bystander killing [44]. The success of second-generation ADCs, such as trastuzumab deruxtecan (T-Dxd) is largely attributed to cleavable linker technology. However, there is no consensus on which linkers are more promising.

Selection of a linker depends on a variety of factors, including the target indication, payload size and toxicity, and the desired pharmacokinetics of the ADC. There are two large categories of linkers currently in use, including cleavable and non-cleavable ones. Cleavable linkers are further subdivided into three categories: peptide linkers (lysosomal enzyme sensitive), such as valine–citrulline linker (or vc linker), disulfide linkers (sensitive to reducing agents like glutathione), and hydrazone linkers (acid-sensitive).

Cleavable linkers have two major advantages compared to the non-cleavable moiety; increased site-specific activation and bystander killing effect. The first FDA-approved ADC, Mylotarg^®^, used a hydrazine-based acid-cleavable linker. One of the most recently approved agents, Trodelvy^®^ (sacituzumab govitecan), is using polyethylene glycol (PEG) spacer technology.

### 2.3. Spacers

Linker technology has its own multiple issues. Most of the payloads are hydrophobic, which limits the drug to antibody ratios (payload aggregates with higher ratios). It also affects serum stability and solubility of the drug, decreasing its half-life [45]. To offset that, several types of spacer molecules were proposed, such as polyethylene glycol (PEG), polar sulfamide, p-aminobenzyl (PAB), and others. Polyethylene glycol polymers are mainly used to offset strong hydrophilic linkers, which, in the case of T-DM1 modification allowed bystander killing, and increased linker stability in the bloodstream [33]. So far, only promising preclinical data is available [46]. In the case of mirvetuximab soravtansine (IMGN853), another approach was used: a short polar sulfamide spacer (HydraSpace™) was paired with a highly hydrophobic payload, which, according to the manufacturer, has increased efficiency of payload conjugation, ADC stability, and its therapeutic index [44].

### 2.4. Bystander Effect

**Figure 4:**
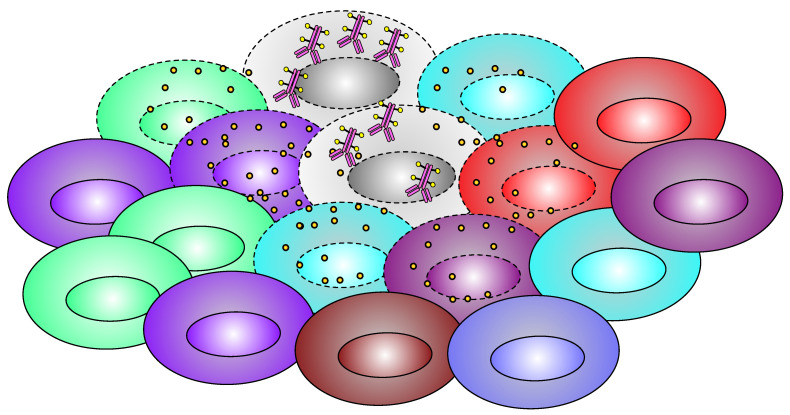
Schematic diagram of bystander effect. Figure Legend: Only one type of cells (gray) is concentrating ADC, but after the cellular death, the payload gets released into the local microenvironment, and the drug diffuses into neighboring cells and causes cell death to them as well.

One of the most difficult aspects of solid tumor treatment is their heterogeneous expression of target antigens (Figure 4). The bystander killing effect on neighboring cells is a key feature of new-generation ADCs. This debated feature of some ADCs is represented by the unintentional payload diffusion from antigen-positive tumor cells to adjacent antigen negative tumor cells. The bystander effect is not exclusive for ADCs and was first observed with conventional therapies such as doxorubicin [47], paclitaxel [48], actinomycin D [49], and radiotherapy [50].

Bystander responses were observed in many cell types, but there are reports suggesting not all cells can generate bystander response. For example, in the case of glioblastoma, cells do not induce a bystander effect if treated with bleomycin but do respond to bleomycin-induced bystander effect from lung adenocarcinoma cells, if incubated together [51].

The magnitude of the bystander effect in case of ADCs depends on several parameters. First, a property of an ADC agent is that to provide the effect, the payload must be lipophilic (otherwise it will not penetrate neighboring cells) [52]. The linker between antibody and payload must also be lysosome-degradable (non-cleavable linkers yield highly charged lysine adducts of drug metabolites, which are unable to diffuse out of cell membrane, resulting in minimal bystander killing) [53]. Another issue is the presence of the target antigen in the environment [54]. As previously shown in the example of fam-trastuzumab deruxtecan and HER-2-negative breast cancer, the antigen does not have to be harbored by tumor cells but needs to be present in the local environment. It has been shown in cell cultures that the magnitude of the bystander effect is directly proportional to the percentage of antigen-positive cells [55]. Given the fact that ADCs are highly selective, they can promote selection of antigen-negative cancer cell subpopulations in the tumor and lose their efficacy with time, or at least significantly decrease their efficacy, and promote loss of target antigen expression.

#### 2.4.1. List of Currently FDA-Approved ADC Therapies and Their Clinical Efficacy in Solid Tumors

Currently, six ADCs are FDA-approved for the treatment of solid tumors, including ado-trastuzumab emtansine (Kadcyla^®^) and trastuzumab deruxtecan (Enhertu^®^), both anti-HER2; enfortumab vedotin (Padcev^®^), targeting Nectin-4; sacituzuzmab govitecan (Trodelvy^®^), active against Trop2; tisotumab vedotin (Tivdak), targeting tissue factor; and mirvetuximab soravtansine (Elahere™), targeting folate receptor alpha (Table 1).

#### 2.4.2. Ado-Trastuzumab Emtansine (Kadcyla^®^), or T-DM1

Ado-trasuzumab emtansine is an ADC consisting of trastuzumab and emtansine (DM1), (a microtubular inhibitor, derivative of maytansine), linked by a stable thioether linker.

T-DM1 was the first FDA-approved ADC for solid tumors. It was granted first approval in February 2013 for HER2+ metastatic or locally advanced breast cancer based on results of the EMILIA trial (NCT00829166), a large phase III randomized trial comparing progression-free survival (PFS) of lapatinib plus capecitabine vs. ado-trastuzumab emtansine [56]. The study enrolled 991 patients with HER2+ advanced breast cancer previously treated with trastuzumab and taxane. The results showed a significant increase in PFS and overall survival (OS) in the T-DM1 group (Table 1). The median PFS was 9.6 months in the T-DM group compared to 6.4 months in the lapatinib plus capecitabine group (hazard ratio (HR) 0.65; 95% CI, 0.55–0.77; *p* < 0.001). The median OS was 30.9 months in the T-DM1 group compared to 25.1 months in the lapatinib plus capecitabine group (HR 0.68; 95% CI, 0.55 to 0.85; *p* < 0.001). The objective response rate (ORR) was higher with T-DM1 (43.6%) compared to lapatinib plus capecitabine (30.8%) (*p* < 0.001).

T-DM1 also had a better safety profile than lapatinib plus capecitabine, with fewer severe adverse events reported (40.8% vs. 57% of grade 3 and above). The most common side effects in the T-DM group were nausea (39%) and fatigue (35%). The most common severe side effects of T-DM1 were thrombocytopenia (12.9%), anemia (2.7%), and liver function test (LFT) elevation (2.9% ALT and 4.3% AST). However, T-DM1 has a higher rate of adverse side effects than trastuzumab alone; especially severe adverse events include hepatotoxicity, cytopenias, anaphylaxis, heart failure, and embryo-fetal toxicity, likely from off-site payload release [57].

Research of T-DM1 has continued, and in May 2019 it was granted approval for use in early-stage breast cancer adjuvant treatment. The FDA approval was based on the KATHERINE trial (NCT01772472), phase III, multicenter, randomized, open label study. The study was comparing efficacy of T-DM1 vs. trastuzumab alone in 1486 early-stage HER2+ breast cancer patients with residual invasive disease after surgery or neoadjuvant systemic therapy (NAST) with taxane plus trastuzumab. After three years, invasive disease or death occurred in 12.2% of patients in the T-DM1 group and 22.2% in the trastuzumab group. Invasive-disease-free survival (IFDS) was significantly higher in the T-DM1 group, 87.7% (85.2–90.2) vs. 76.9% (73.7–80.1) in trastuzumab alone (HR of 0.50, 95%CI 0.39–0.63, *p* < 0.001). In other words, T-DM1 showed 50% risk reduction for 3-year invasive disease recurrence compared to trastuzumab. The rate of distant metastases was also lower in the T-DM1 group (10.5%) compared to 15.9% trastuzumab alone [22].

The rate of adverse events was higher in the T-DM1 group compared to trastuzumab group (98.8% vs. 93.3% overall and 25.7% vs. 15.7% grade 3 and above, respectively). All side effects were common in T-DM1 group except for hot flashes (12.8% T-DM1 vs. 20.3% trastuzumab group). Most common adverse events were fatigue (49.5% vs. 33.8%), nausea (41.6% vs. 13.1%), thrombocytopenia (28.5% vs. 2.4%), ALT rise (28.5% vs. 5.6%), AST rise (23.1% vs. 5.7%), headache (28.4% vs. 16.9%), arthralgia (25.9% vs. 20.6%), epistaxis (21.5% vs. 3.5%), neuropathy (18.6% vs. 6.9%), constipation (17% vs. 8.2%), myalgia (15.4% vs. 11.1%), hot flashes (12.8% vs. 20.3%). Most common grade ≥ 3 adverse events in T-DM1 group were thrombocytopenia (5.7% vs. 0.3%), hypertension (2% vs. 1.2%), radiation-related skin injury (1.4% vs. 1%), peripheral neuropathy (1.4% vs. 0%), neutropenia (1.2% vs. 0.7%), hypokalemia (1.2% vs. 0.1%), fatigue (1.1% vs. 0.1%), anemia (1.1% vs. 0.1%).

Trastuzumab is also approved for HER2-positive gastric adenocarcinoma. However, in contrast to breast cancer, T-DM1 has failed to show superiority to taxanes in HER2-positive previously treated advanced gastric cancer patients. The adaptive phase II/III GATSBY trial (NCT01641939) [58] showed no difference in OS or PFS for ado-trastuzumab emtansine compared with taxane treatment.

#### 2.4.3. Trastuzumab Deruxtecan (Enhertu^®^) or T-Dxd

Trastuzumab deruxtecan (trade name Enhertu) is an antibody-drug conjugate comprised of the humanized monoclonal antibody trastuzumab covalently linked to the topoisomerase I inhibitor deruxtecan. It is also known as T-Dxd or fam-trastuzumab deruxtecan-nxki, and it is another trastuzumab-based ADC first approved by the FDA in December 2019 for the treatment of HER2-positive unresectable metastatic breast cancer [59]. In 2021, T-Dxd became the first ADC approved for HER2-positive gastric cancer. It is also approved for *HER2*-mutant NSCLC as well as HER2 low (1+ or 2+ by IHC) breast cancer. T-Dxd is a trastuzumab-based ADC linked to topoisomerase I inhibitor DXd via a lysosome-cleavable linker, while T-DM1 is linked with emtansine (microtubular inhibitor) via a non-cleavable linker. T-Dxd appears to be superior to chemotherapy and T-DM1 in most indications, showing effectiveness in HER2-positive gastric and NSCLC, as well as HER2-low tumors. The difference in linker technology is thought to be primarily responsible for the difference in efficacy between these two agents [60].

- Breast Cancer

In a two-part, open-label, single-group, multicenter, phase 2 trial DESTINY-Breast01 (n = 184) (NCT03248492), T-Dxd was evaluated in adults with HER2-positive metastatic breast cancer who had received previous treatment with T-DM1. ORR was 60.9% (95% CI, 53.4–68.0), median response duration was 14.8 months (95% CI, 13.8 to 16.9), and the median duration of PFS was 16.4 months (95% CI, 12.7 to not reached). During the study, the most common adverse events of grade 3 or higher were neutropenia (20.7% of the patients), anemia (8.7%), and nausea (7.6%). On independent adjudication, the trial drug was associated with interstitial lung disease in 13.6% of the patients (grade 1 or 2, 10.9%; grade 3 or 4, 0.5%; and grade 5, 2.2%), which led to an FDA black-box warning on pulmonary complications, including interstitial lung disease and pneumonitis, as well as embryo-fetal toxicity.

On 5 August 2022, the FDA approved T-Dxd for patients with unresectable or metastatic HER2-low (HER2 1+ or 2+ by IHC) breast cancer. The approval was based on the results of the DESTINY-Breast04 trial, (NCT03734029), which included 557 patients with HER2-low breast cancer [7]. Patients were randomized to receive T-Dxd or physician’s chemotherapy choice. The trial included two cohorts: 494 hormone receptor-positive patients and 63 hormone receptor-negative patients. The median PFS was significantly higher in the T-Dxd group than the physician’s choice group (HR 0.51, 95% CI, 0.40 to 0.64; *p* < 0.001) for the hormone receptor-positive cohort and 0.46 (95% CI, 0.24 to 0.89) for the hormone receptor-negative cohort. The median OS was also higher in the T-Dxd group compared to the physician’s choice group for the hormone receptor-positive group (HR 0.64, 95% CI, 0.48 to 0.86; *p* = 0.003) compared to the hormone receptor-negative cohort (HR 0.48, 95% CI, 0.24 to 0.95). The percentage of patients with a confirmed objective response was 52.6% (95% CI, 47.0 to 58.0) in the T-Dxd group and 16.3% (95% CI, 11.0 to 22.8) in the physician’s choice group for the hormone receptor-positive cohort. The median duration of response was 10.7 months in the T-Dxd group and 6.8 months in the physician’s choice group.

The incidence of serious adverse events was similar between the two groups (27.8% in T-Dxd vs. 25.0% in physician’s choice), while the incidence of adverse events of grade 3 or higher was elevated in the physician’s choice group (67.4% vs. 52.6%). Drug-related deaths were higher in the T-Dxd group (3.8% vs. 2.9% in physician’s choice), with pneumonitis being the most common cause (n-2, 0.5%) [61].

- Gastric Cancer

In DESTINY-Gastric01 trial (NCT03329690), T-Dxd demonstrated significant efficacy in treating adult patients with locally advanced or metastatic HER2-positive gastric or gastroesophageal adenocarcinoma who have previously received trastuzumab-based therapy. The study evaluated 188 patients who had progressed on at least two prior regimens, including trastuzumab, fluoropyrimidine-, and a platinum-containing chemotherapy. T-Dxd group demonstrated a median OS of 12.5 (9.6–14.3) months vs. 8.4 (9.6–10.7) months in the irinotecan/paclitaxel arm (HR 0.59, 95% CI, 0.39 to 0.88, *p* = 0.0097) and a confirmed objective response rate of 40.5% (31.8–49.6) vs. 11.3% (4.7–21.9), with duration of response of 11.3 months vs. 3.9 months, respectively. The most common adverse reactions were similar to previously reported, such as nausea (63%), decreased neutrophil count (63%), decreased appetite (60%), anemia (58%), thrombocytopenia (58%), leukopenia (38%), malaise (34%), diarrhea (32%), constipation (24%), fever (24%), alopecia (22%), fatigue (22%), and lymphopenia (22%) [6]. In January 2021, the FDA granted approval of T-Dxd use in patients with locally advanced or metastatic HER2-positive gastric or gastroesophageal adenocarcinoma who have received a prior trastuzumab-based regimen [62].

- Lung Cancer

T-Dxd was also granted accelerated FDA approval for metastatic treatment-refractory NSCLC in August 2022, after analysis of the DESTINITY-Lung01 trial (NCT03505710) results, a multicenter, international, phase II study of patients with metastatic *HER2*-mutant NSCLC that was refractory to standard treatment. A total of 91 patients were enrolled, with a median follow-up of 13.1 months. The primary outcome of centrally confirmed objective response was observed in 55% of patients (95% CI, 44 to 65), and the median duration of response was 9.3 months (95% CI, 5.7 to 14.7). Median PFS was 8.2 months (95% CI, 6.0 to 11.9), and median OS was 17.8 months (95% CI, 13.8 to 22.1). However, the safety profile included grade 3 or higher drug-related adverse events in 46% of patients, with neutropenia being the most common event (19%). Adjudicated drug-related interstitial lung disease occurred in 26% of patients and resulted in death in two patients [62].

## 3. Enfortumab Vedotin (Padcev^®^)

Enfortumab vedotin (trade name Padcev) is an antibody-drug conjugate used for the therapy of urothelial cancer. It is a nectin-4-directed antibody and microtubule inhibitor conjugate. Enfortumab refers to the monoclonal antibody part, and vedotin refers to the payload drug and the linker.

Based on results of the EV-301 trial (NCT03474107), enfortumab vedotin was approved by the FDA in July 2021 for the treatment of adult patients with locally advanced or metastatic urothelial cancer. EV-301 was a randomized, multicenter trial involving 608 patients with locally advanced or metastatic urothelial cancer who had previously received a programmed cell death-1 (PD-1 or PD-L1 inhibitor and platinum-based chemotherapy. Treatment with enfortumab vedotin resulted in a significantly longer OS compared to chemotherapy (HR 0.70, 95% CI, 0.56 to 0.89; *p* = 0.001). The median OS was 12.8 months (95% CI, 10.58 to 15.21) in the enfortumab vedotin group vs. 8.9 months (95% CI, 8.05 to 10.74) in the chemotherapy group. Treatment with enfortumab vedotin also resulted in a significantly longer PFS (HR 0.62, 95% CI, 0.51 to 0.75; *p* < 0.001) and a higher confirmed overall response rate (40.6%, 95% CI, 34.9 to 46.5) when compared to the chemotherapy group (17.9%, 95% CI, 13.7 to 22.8, *p <* 0.001). A complete response was observed in 4.9% of the patients in the enfortumab vedotin group vs. 2.7% of the patients in the chemotherapy group. Disease control was observed in 71.9% (95% CI, 66.3 to 77.0) and 53.4% (95% CI, 47.5 to 59.2) of patients, respectively *(p <* 0.001) [23].

## 4. Sacituzuzmab Govitecan (Trodelvy^®^)

Sacituzumab govitecan is an ADC consisting of a humanized anti-Trop-2 mAb coupled with the payload topoisomerase inhibitor (SN-38, active metabolite of irinotecan).

- Triple-negative breast cancer

Trophoblast cell surface antigen 2 (Trop-2) is a transmembrane glycoprotein involved several oncogenic pathways (cyclin E/D1, MAPK, RAF-FOXM1, NFkB, b-catenin) [63]. Sacituzumab govitecan was granted its first FDA approval on April 2021, following results of pivotal ASCENT trial (NCT02574455), a randomized, phase III study conducted in patients with metastatic triple-negative breast cancer who had received at least two prior chemotherapies, including at least one in the metastatic setting. Of the 529 patients enrolled, 468 did not have known brain metastases at baseline. The objective response rate was 35% in the sacituzumab govitecan group and 5% with chemotherapy. Sacituzumab govitecan compared to single-agent chemotherapy significantly improved both median PFS (5.6 vs. 1.7 months, respectively; HR 0.39; *p* < 0.0001) and median OS (12.1 vs. 6.7 months, respectively; HR 0.48; *p* < 0.0001). The OS rate at 24 months was 22.4% (95% CI, 16.8 to 28.5) in the sacituzumab govitecan arm and 5.2% (95% CI, 2.5 to 9.4) in the chemotherapy arm.

Important treatment-related grade 3 and higher adverse events with sacituzumab govitecan vs. chemotherapy were diarrhea (11% vs. 0.4%), neutropenia (52% vs. 33%), anemia (8% vs. 5%), and febrile neutropenia (6% vs. 2%). There was no severe (grade 3) neuropathy and only one case of grade 3 interstitial lung disease reported in the SG arm. No patient experienced a treatment-related death with sacituzumab govitecan, and there was one treatment-related death with chemotherapy due to neutropenic sepsis.

- Hormone receptor-positive, HER2-negative breast cancer

Later on, in February 2023, sacituzumab govitecan was FDA-approved for patients with unresectable locally advanced or metastatic HR-positive, HER2-negative breast cancer who have received endocrine-based therapy and at least two additional systemic therapies in the metastatic setting. The efficacy of sacituzumab govitecan was evaluated in the TROPiCS-02 study (NCT03901339), a global, multicenter, open-label, phase III study that randomly assigned 543 patients to sacituzumab govitecan vs. physicians’ choice of chemotherapy (eribulin, capecitabine, gemcitabine, or vinorelbine). The sacituzumab govitecan arm demonstrated a median PFS of 5.5 months (95% CI, 4.2 to 7.0) compared to 4 months (95% CI, 3.1 to 4.4) in the single-agent chemotherapy arm (HR 0.661, 95% CI, 0.529 to 0.826 *p* = 0.0003). The OS was also improved in patients receiving sacituzumab govitecan, with a median OS of 14.4 months (95% CI, 13.0 to 15.7) compared to 11.2 months (95% CI, 10.1 to 12.7) in the single agent chemotherapy arm (HR 0.789; 0.646 to 0.964; *p* = 0.0200). The most common adverse events included leukopenia (88%), neutropenia (83%), and diarrhea (62%) [26].

Despite FDA approvals, concerns have been raised regarding whether sacituzumab govitecan acts as a conventional ADC or a prodrug form of irinotecan, mainly because it has shown efficacy even in Trop2-negative tumors, such as small cell lung cancer. It has been suggested that protease-cleavable linker allows partial systemic release of the payload (much higher than in other ADCs), irrespective of antigen presence and thus acting as a prodrug of irinotecan. There are some pharmacokinetic data supporting that hypothesis [11].

## 5. Tisotumab Vedotin (Tivdak^®^) or TV

Tisotumab vedotin (trade nameTivdak) is an antibody–drug conjugate utilized to treat cervical cancer. It is a combination of tisotumab, a monoclonal antibody against tissue factor, and monomethyl auristatin E, a potent cell division inhibitor. Tisotumab vedotin has demonstrated clinically meaningful and durable antitumor activity with a manageable and tolerable safety profile in women with previously treated recurrent or metastatic cervical cancer. The FDA granted accelerated approval to tisotumab vedotin in September 2021 [64] based on the innovaTV 204/GOG-3023/ENGOT-cx6 trial (NCT03438396), an open-label, multicenter, single-arm study that enrolled 101 patients with recurrent or metastatic cervical cancer who had received no more than two prior systemic regimens. The confirmed objective response rate was 24% (95% CI, 16 to 33), with seven complete responses and 17 partial responses. The most common treatment-related adverse events in the tisotumab vedotin were alopecia (38%), epistaxis (30%), nausea (27%), conjunctivitis (26%), fatigue (26%), and dry eye (23%) [27].

## 6. Mirvetuximab Soravtansine (Elahere™)

Mirvetuximab soravtansine is an ADC consisting of a humanized anti-folate receptor alpha (FRα) mAb linked to the tubulin-disrupting maytansinoid DM4. It was granted accelerated approval by the FDA in November 2022, for adult patients with folate receptor alpha (FRα) positive, platinum-resistant epithelial ovarian, fallopian tube, or primary peritoneal cancer who have received one to three prior systemic treatment regimens.

The approval was based on preliminary results of the SORAYA study, also known as Study 0417 (NCT04296890), a single-arm trial of 106 patients with FRα positive, platinum-resistant epithelial ovarian, fallopian tube, or primary peritoneal cancer (n = 104). The confirmed overall response rate was 31.7% (95% CI, 22.9 to 41.6) and median duration of response was 6.9 months (95% CI. 5.6 to 9.7). The FDA has also approved specific companion sensitivity assay VENTANA FOLR1 (FOLR-2.1) RxDx Assay (Ventana Medical Systems, Inc., Oro Valley, Arizona, USA), a laboratory test designed to detect FRα protein.

Safety population analysis (n-106) showed a frequency of treatment-related adverse events (TRAEs) of 86% (n-91), with severe events in 30% of participants and 11% severe TRAE, leading to dose reduction in 20% of patients, dose delay in 33% of patients, drug discontinuation in 9% of cases, and one treatment-related death from respiratory failure in metastatic lung involvement complicated by diffuse alveolar hemorrhage and idiopathic pulmonary fibrosis. Six other patients died during the study, four from disease progression and two from unrelated adverse events [28,65,66].

### 6.1. Solid Tumor ADCs in the Pipeline

Aside from the ongoing series of DESTINY trials for T-Dxd, there are several other agents currently undergoing phase III clinical trials on clinicaltrials.gov (Table 2), mostly HER2-based ADCs. As of April 2023, there are at least 16 clinical trials with active enrollment and no published results. A short list of phase III agents (only for solid tumors) is provided below.

- RC48-ADC, also known as disitamab vedotin, is a newly developed ADC drug targeting HER2. It is comprised of hertuzumab coupling MMAE via a cleavable linker. It has demonstrated promising anti-tumor activity in pre-clinical and early clinical studies (phase II, single arm, n-43), showing ORR of 51.2% (35.5–66.7%) as a second line treatment of patients with HER2+ locally advanced or metastatic urothelial cancer previously treated platinum-containing chemotherapy. The agent has already been approved for use in China in June 2021 [67,68]. Currently, there are three active phase III clinical trials for RC-48 for its use in HER2-positive breast cancer, HER2-low metastatic breast cancer, and urothelial carcinoma.
- MRG002 is a novel HER2-targeting ADC with potent antitumor activity against HER2-positive solid tumors. It is composed of a modified (hyper-fucosylated) trastuzumab, MMAE payload and a cleavable vc-linker, similar to T-DM1, but with favorable toxicity profile according to preclinical data [69]. A phase I trial in HER-2-positive breast carcinoma showed ORR of 34.7% and DCR of 75.5%, with n-17 PR, 20 SD, and 12 PD [70]. There are 11 ongoing clinical trials for breast, lung, and gastric carcinomas, including two phase III trials: NCT04924699 for HER2-positive unresectable locally advanced or metastatic breast cancer and NCT05754853 for HER-2-positive unresectable or advanced metastatic urothelial cancer.
- ARX788 is an anti-HER2 ADC that utilizes a unique nonnatural amino acid-enabled conjugation technology and a noncleavable drug-linker amberstatin (AS269), a potent tubulin inhibitor. The special drug-linker structure is designed to increase drug serum stability and to decrease off-site activation. In preclinical data on mice, ARX788 showed a half-life of 12.5 days [71]. It received fast track designation from the FDA in early 2021 based on phase I trial (CTR20171162/ACE-Breast-01) data in HER-2 positive breast cancer, with ORR of 19/29 or 66% (45.7–82.1%) [72]. There is currently ongoing phase II trial ACE-Breast03 (NCT04829604). ARX788 is also being studied for advanced gastric and gastrojejunal junction adenocarcinoma patients, with preliminary phase I trial data showing ORR of 45.5% (9/23) [73].
- SYD985, also known as trastuzumab duocarmazine, is another HER2-targeting ADC with a cleavable linker-duocarmycin payload. As mentioned in the payload section, duocarmazine-based ADCs have been extensively studied over the past 50 years, but none of them have been FDA approved in oncology so far. Trastuzumab duocarmazine received FDA fast track designation in 2018 based on early clinical data [74,75]. It is currently undergoing a phase III trial (TULIP/NCT03262935, n-437), where trastuzumab duocarmazine was compared to physician’s choice of treatment in patients with heavily pre-treated HER-2 locally advanced or metastatic breast cancer. Primary outcome analysis results published in 2021 showed a difference in PFS of 7.0 months (5.4–7.2) for SYD985 compared 4.9 months (4.0–5.5) for PC [76].
- XMT-1536 also known as upifitamab rilsodotin (UpRi), is a first-in-class dolaflexin (dolastatin/MMAE family) ADC targeting sodium-dependent phosphate transporter NaPi2b, linked with proprietary DolaLock payload auristatin F-hydroxypropylamide (AF-HPA). F-hydroxyprolylamide was designed to be lipophilic with intracellular metabolic conversion into a less lipophilic metabolite to balance bystander killing and intracellular trapping [77]. NaPi2b is broadly expressed in solid tumors such as serous epithelial ovarian cancer and NCLSC. Some limited clinical data released by the company showed an ORR of 32% and disease control rate of 74% in ovarian cancer patients (n = 31) with NaPi2b overexpression [78].
- Datopotamab deruxtecan, also known as Dato-DXd, is another Trop2-directed ADC being jointly developed by AstraZeneca and Daiichi Sankyo. It is made up of a humanized anti-Trop2 IgG1 mAb attached to a deruxtecan payload via a stable tetrapeptide-based cleavable linker. The agent is being studied in a series of TROPION trials series. Data from TROPION-PanTumor01 (NCT03401385) has been published. A total of 24 patients with triple negative breast cancer were treated with Dato-Dxd, with an overall response rate of 43% and a disease control rate of 95%. The most common side effects (any grade) were nausea, stomatitis, fatigue, and vomiting. Thirty-three percent of patients experienced grade 3 or higher treatment-emergent adverse events. Yet, no patients discontinued treatment, and no cases of drug-related interstitial lung disease were reported [79]. More recently, AstraZeneca made an announcement on their website that Dato-DXd met dual primary endpoint in the TROPION-Lung01 phase III trial, but no details have been published yet [80].

### 6.2. ADC Limitations and Side Effects

Antibody–drug conjugates are widely used in hematological malignancies and have not been that successful in solid tumors. One of the key differences is solid tumor complex microarchitecture and matrix microenvironment. In hematological tumors, there is a large number of circulating tumor cells in the bloodstream with somewhat homogenous antigen expression, in contrast to a highly heterogeneous solid tumor microenvironment [81]. While hematological cells in the circulation are directly exposed to ADC, in solid tumors, both extracellular matrix (stroma) and tumor cells themselves impede antibody diffusion [82,83]. Since ADCs are relatively large molecules, their distribution in the tumor may be uneven (lack of tissue penetration), which may cause variable clinical effects of the drug. In order to overcome these barriers, ADCs for solid tumors should have long plasma half-life, chemical stability, and relatively low molecular mass, which is difficult to achieve [84]. On the positive side, antigen heterogeneity and low issue penetrance can be somewhat overcome with the bystander effect and payload tissue accumulation; however, this hypothesis is technically difficult to prove [17,85].

Despite great potential, ADC use in solid tumor oncology remains limited. There are three significant barriers towards widespread use of ADCs. First, the complexity of ADC production and research. As mentioned previously, payloads need to be extremely cytotoxic, pushing towards search of exotic agents, e.g., sea hare peptide derivatives (MMAE). Hydrophobic and hydrophilic properties of the payload, linker, and the antibody need to be balanced, which is complicated.

The balance between tumor tissue and systemic payload release is another major concern. As shown in Table 3, some ADCs have toxicities akin to chemotherapy (e.g., T-Dxd). Though lower-dose regimens might ameliorate some of these side effects, ADCs can also recognize non-tumor cells carrying the target antigen, leading to “on-target off-tumor cytotoxicity”. Clinical efficacy assessment of ADCs is more complicated because of off-target payload release and risks of confounding with payload systemic effects [11]. More studies of linkers and spacers, robust and standardized pharmacokinetic studies, and appropriate comparator choices may mitigate risks of confounding.

The complex biology of ADCs also possesses inherent risks of multiple resistance mechanisms: lowering target antigen expression, altering ADC intracellular metabolism, efflux of payload [87]. From a tumor biology perspective, the concern is a lack of actionable targets. NGS has been widely recognized in solid tumors as a way to identify clinically actionable genomic alterations [88,89], especially for tissue agnostic agents [90,91]. It has been reported previously that the rate of such alterations may vary from 40 to 94% [92]. Unfortunately, not all patients have targetable mutations. The rate of actionable genomic alterations may vary from <10% to almost 90%, depending on the trial [93,94,95,96]. It has been proposed that transcriptome sequencing may provide better data for precision therapy, and several studies have shown varying degrees of utility of RNA expression in the clinic [97,98,99,100]. Another approach is matching scores based on mutational footprints of cancer-driving mutations, with the higher the score, the better the chances of response [94,95,96,101].

A final issue may be the cost of ADCs. These are expensive drugs to develop and to administer. So far, none of the ADCs approved for solid tumors can be considered cost-effective. However, cost effectiveness is highly dependent on the local markets and may vary from country to country [8,102,103].

We believe that transparency, active research funding, and promoting open international competition in the field may help with the cost issue. Overall, unfortunately, the cost issue is very complex and does not solely belong to ADCs and does not have a simple solution [104,105].

## 7. Conclusions

In summary, ADCs have emerged as a promising cancer treatment strategy due to their ability to deliver potent cytotoxic payloads selectively to cancer cells, minimizing the side effects associated with traditional chemotherapy. However, ADCs still face several challenges, including identifying suitable targets, optimizing drug–antibody ratios, minimizing non-specific toxicity, overcoming drug resistance, and achieving cost-effectiveness. Despite these challenges, ADCs continue to hold great potential as an effective cancer therapy, and ongoing research aims to improve their efficacy, reduce toxicity, and increase cost-effectiveness, ultimately improving outcomes.

## Figures and Tables

**Table 1:** FDA-approved ADCs for solid tumors.

Drug	Target	Payload	Linker	DAR	Indication of Approval	Date of FDA Approval	Outcomes	Ref.
Ado-trastuzumab emtansine (Kadcyla) or T-DM	HER2	Emtansine (DM1) *	Non-cleavable	3.5	HER2-positive breast cancer, late stage (EMILIA (phase III)	February 2013	ORR 43.6% vs. lapatinib + capecitabine 30.8%, *p* < 0.001, CR 1%, PR 42.6%, PFS 12.6 months (95% CI 8.4–20.8) vs. 6.5 months (95% CI 5.5–7.2)	[21]
					HER2-positive breast cancer, early stage, adjuvant treatment (KATHERINE Trial)	May 2019	3-year IDFS (T1DM vs. trastuzumab), 87.7% (85.2–90.2) vs. 76.9% (73.7–80.1), HR 0.51 (0.40–0.66), 3-year disease-free survival—87.4% (84.9–89.9) vs. 76.9 (73.6–80.1), overall survival 5.7% vs. 7.5% (*p* = 0.08)	[22]
Enfortumab vedotin, EV (Padcev)	Nectin-4	MMAE *	Enzyme-cleavable	3.8	Advanced urothelial cancer (EV-301 Trial)	December 2020	EV vs. chemotherapy, ORR 40.6% (34.9–46.54) vs. 17.9% (13.7–22.76), *p* < 0.001; CR 4.9% vs. 2.7%, PR 35.8% vs. 15.2%, SD 31.3% vs. 35.5%, PD 15.3% vs. 28%	[23]
Trastuzumab deruxtecan/T-DXd (Enhertu)	HER2	Deruxtecan **	Enzyme-cleavable	8	HER2-positive unresectable or metastatic breast cancer following two or more prior anti-HER2-based regimens (DESTINY-Breast01 trial)	December 2019	Single arm, ORR 60.9% (53.4–68), PFS 16.4 months (12.7-not reached), detailed response rates (6.4 mg/kg–7.4 mg/kg): CR 4.2–4.8%, PR 64.6–81%, SD 29.2–14.3%	[24]
					HER-2-positive advanced gastric or gastrojejunal junction adenocarcinoma (DESTINY-Gastric01 trial)	December 2019	T-Dxd vs. chemotherapy, confirmed ORR 43% (34–52) vs. 12 (5–24), CR 8% vs. 0, PR 34% vs. 12%, SD 43% vs. 50%, PR 12% vs. 30%	[6]
					HER2-low unresectable or metastatic breast cancer after 2 or more HER-2 regimens, or rapid (<6 months) recurrence (DESTINY-Breast04)	5 August 2022	T-Dxd vs. chemotherapy (all patients), ORR 52.3% (47.1–57.4) vs. 16.3 (11.3–22.5), CR 3.5% vs. 0.5%, PR 48.8% vs. 15.8%	[7]
					HER2+ unresectable advanced non-small-cell lung cancer (NSCLC) (DESTINY-Lung02 trial)	11 August 2022	T-Dxd, ORR 55% (44–65%), CR 1%, PR 54%, SD 37%, PD 3%	[5]
Sacituzumab Govitecan (Trodelvy)	Trop2	SN-38 ** (active metabolite of irinotecan)	Acid-cleavable	7.6	Previously treated metastatic triple negative breast cancer (ASCENT Trial)	April 2020	SG vs. TPC, PFS 4.8 months (4.1–5.8) vs. 1.7 (1.5–2.5), ORR—31% vs. 4%, CR 4% vs. 1%, PR 27% vs. 3%, SD at 6 months 9% vs. 4%, PD 24% vs. 38%	[25]
					HR+/HER2− metastatic breast cancer patients who had received prior endocrine-based therapy and at least two chemotherapies (TROPiCS-02 trial, Phase II)	3 February 2023	SG (n = 272) vs. TPC (n = 271), PFS 5.5 months (4.2–7.0) vs. 4 months (3.1–4.4), OS 14.4 months (13–15.7), 11.2 months (10.1–12.7),	[26]
Tisotumab vedotin (Tivdak)	Tissue factor (CD 142)	MMAE *	Enzyme-cleavable	4	Recurrent or metastatic cervical cancer (innovaTV 204 trial)	September 2021	Single arm, ORR 24% (16–33%), CR 7%, PR 17%, SD 49%, PD 24%	[27]
Mirvetuximab soravtansine	folate receptor alpha (mirvetuximab)	DM4 *	Cleavable	3–4	folate receptor alpha (FRα)-positive, platinum-resistant epithelial ovarian, fallopian tube, or primary peritoneal cancer, who have received one to three prior systemic treatment regimens (Study 0417)	14 November 2022	Single arm (n = 96 by BICR), ORR 30.2% (21.3–40.4%), CR (6.3%), PR (24%), SD 56.3%, PD 9.4%	[28]

*—antimicrotubular agent; **—topoisomerase inhibitor, TPC—treatment of physician’s choice, IDFS—invasive disease-free survival, PFS—progression-free survival, ORR—objective response rate; CR, complete response; PD—progressive disease; PR—partial response; SD—stable disease; 95% confidence intervals provided in parentheses, MMAE—Monomethyl auristatin E, GD2—disialoganglioside (cell surface glycolipid, present at low concentrations on skin, neural and PNS cells, overexpressed on neuroblastoma cells). BICR—blinded independent central review. Response rates are provided according to RECIST 1.1 criteria.

**Table 2:** Current phase III active ADC clinical trials.

NCT Number	Title	Sponsor/Collaborators	Target	Payload	Linker
NCT04714190	A Study of RC48-ADC in Local Advanced or Metastatic Gastric Cancer With the HER2-Overexpression	RemeGen Co., Ltd., Yantai, Shandong, China	HER2 (Hertuzumab)	MMAE	vc-cleavable
NCT04400695	A Study of RC48-ADC for the Treatment of Locally Advanced or Metastatic Breast Cancer With Low Expression of HER2				
NCT03500380	A Study of RC48-ADC Administered Intravenously to Patients With HER2-Positive Metastatic Breast Cancer With or Without Liver Metastases				
NCT05302284	A Study of RC48-ADC Combined With Toripalimab For First-line Treatment of Urothelial Carcinoma				
NCT05329545	Upifitamab Rilsodotin Maintenance in Platinum-Sensitive Recurrent Ovarian Cancer (UP-NEXT)	Mersana Therapeutics Cambridge, MA USA|GOG Foundation| (ENGOT)	NaPi2b	AF-HPA (DolaLock^®^)	Protease-cleavable
NCT04595565	Sacituzumab Govitecan in Primary HER2-negative Breast Cancer	German Breast Group| (GEICAM)|ETOP IBCSG Partners Foundation|Cancer Trials Ireland|UNICANCER	Trop-2	SN38 (irinotecan)	Protease cleavable
NCT05609968	Study of Pembrolizumab (MK-3475) Monotherapy Versus Sacituzumab Govitecan in Combination With Pembrolizumab for Participants With Metastatic Non-small Cell Lung Cancer (NSCLC) With Programmed Cell Death Ligand 1 (PD-L1) Tumor Proportion Score (TPS), (MK-3475-D46)	Merck Sharp & Dohme LLC Rahway, NJ, USA|Gilead Sciences Foster City, CA, USA			
NCT05622890	A Single-arm Clinical Trial of IMGN853 in Chinese Adult Patients With Platinum-resistant, Epithelial Ovarian Cancer	Hangzhou Zhongmei Huadong Pharmaceutical Co., Ltd. Hangzhou, China	FRα	DM4	Cleavable
NCT05445778	Mirvetuximab Soravtansine With Bevacizumab Versus Bevacizumab as Maintenance in Platinum-sensitive Ovarian, Fallopian Tube, or Peritoneal Cancer (GLORIOSA)	ImmunoGen, Inc. Waltham, MA, USA|GOG Foundation			
NCT04924699	A Study of MRG002 in the Treatment of Patients With HER2-positive Unresectable Locally Advanced or Metastatic Breast Cancer	Shanghai Miracogen Inc. Shanghai, China	HER2 (fucosylated trastuzumab)	MMAE	Vc-cleavable
NCT05629585	A Study of Dato-DXd With or Without Durvalumab Versus Investigator’s Choice of Therapy in Patients With Stage I-III Triple-negative Breast Cancer Without Pathological Complete Response Following Neoadjuvant Therapy (TROPION-Breast03)	AstraZeneca, Wilmington, DE, USA|Daiichi Sankyo, Inc. Tokyo, Japan |SWOG Clinical Trials Partnerships	Trop2 (Datopotamab)	Deruxtecan (Dxd)	Tetrapeptide, cleavable
NCT05374512	A Study of Dato-DXd Versus Investigator’s Choice Chemotherapy in Patients With Locally Recurrent Inoperable or Metastatic Triple-negative Breast Cancer, Who Are Not Candidates for PD-1/PD-L1 Inhibitor Therapy (TROPION-Breast02)	AstraZeneca Wilmington, DE, USA |Daiichi Sankyo, Inc. Tokyo, Japan			
NCT05687266	Study of Datopotamab Deruxtecan (Dato-DXd) in Combination With Durvalumab and Carboplatin for First-Line Treatment of Patients With Advanced NSCLC Without Actionable Genomic Alterations	AstraZeneca Wilmington, DE, USA			
NCT04494425	Study of Trastuzumab Deruxtecan (T-DXd) vs. Investigator’s Choice Chemotherapy in HER2-low, Hormone Receptor Positive, Metastatic Breast Cancer	AstraZeneca Wilmington, DE, USA |Daiichi Sankyo Company, Limited 3-5-1 Nihonbashihoncho, Chuo-ku, Tokyo, Japan	HER2 (Trastuzumab)	Deruxtecan (Dxd)	Vc-cleavable
NCT05426486	A Study of ARX788 Combined With Pyrotinib Maleate Versus TCBHP (Trastuzumab Plus Pertuzumab With Docetaxel and Carboplatin) as Neoadjuvant Treatment in HER2-positive Breast Cancer Patients	Caigang Liu|NovoCodex Biopharmaceuticals Co., Ltd. Yuecheng, Zhejiang, China |Jiangsu HengRui Medicine Co., Ltd. Lianyungang, Jiangsu Province, China |Shengjing Hospital	HER2	MMAF (AS269)	para-acetylphenylalamine (pAcF); non-cleavable
NCT03262935 *	SYD985 vs. Physician’s Choice in Participants With HER2-positive Locally Advanced or Metastatic Breast Cancer	Byondis B.V. Nijmegen, Netherlands	HER2 (Trastuzumab)	Duocarmazine	Vc-cleavable

*—not currently recruiting new patients, NaPi2b—sodium-dependent phosphate transport protein, AF-HPA—auristatin F-hydroxypropylamide, FRα—folate receptor alpha, vc-cleavable—valine–citrulline protease cleavable linker.

**Table 3:** ADC-associated adverse events.

Agent	Trial (n-Treatment Arm)	Most Common Side Effects (Any Grade)	Grade ≥ 3 Adverse Events	Agent Interruption/Discontinuation	Grade 5 (Deaths)	Ref.
T-DM1	KATHERINE trial (n = 740)	Any adverse event 98.8%, fatigue 49.5%, nausea 41.6%, thrombocytopenia 28.5%, increased AST 28.4%, headache 28.4%, arthralgia 25.9%, radiation-related skin injury 25.4%, increased ALT 23.1%, epistaxis 21.5%, peripheral sensory neuropathy 18.6%, constipation 17%, myalgia 15.4%, hot flashes 12.8%	Any event 25.7%; thrombocytopenia 5.7%, hypertension 2.0%, radiation-related skin injury 1.4%, peripheral sensory neuropathy 1.4%, neutropenia is 1.2%, hypokalemia 1.2%, fatigue 1.1%, anemia 1.1%	Any event 18%; thrombocytopenia 4.2%, hyperbilirubinemia 2.6%, elevated AST 1.6%, elevated ALT 1.5%, peripheral neuropathy 1.5%, decreased ejection fraction 1.2%	0.1%	[22]
	TH3RESA (n = 403)	Nausea 35%, fatigue 29%, headache 24%, vomiting 18%, asthenia 18%, pyrexia 20%, epistaxis 16%, arthralgia 15%, thrombocytopenia 15%, constipation 22%, cough 19%, lack of appetite 16%, myalgia 11%, increased AST 10%, dyspnea 9%, anemia 8%, abdominal pain 6%, rash 6%, neutropenia 5%, and leukopenia 2%.	Any event 40%; thrombocytopenia 5%, increased AST 2%, anemia 1%, neutropenia 2%, fatigue 2%, dyspnea 2%, increased ALT 2%.	15% Discontinued, 13% dose reduction	0.6%	[86]
	EMILIA (n = 397)	Any event 94.9%, event-specific rates: nausea 39.2%, fatigue 35.1%, diarrhea 23.3%, thrombocytopenia 28%, elevated AST 22.4%, elevated ALT 16.9%, vomiting 19%, hypokalemia 8.6%, neutropenia 5.9%, mucosal inflammation 6.7%, palmar plantar erythrodysesthesia 1.2%	Thrombocytopenia 12.9%, elevated ALT 4.3%, elevated ALT 2.9%, anemia 2.7%, fatigue 2.4%, hypokalemia 2.2%, neutropenia 2%, diarrhea 1.6%, vomiting 0.8%, nausea 0.8% mucosal inflammation 0.2%	T-Dm was discontinued in 5.9% of patients, mainly due to thrombocytopenia	0. 2%	[56]
T-Dxd	DESTINY-Breast01 (n = 184)	Any adverse event 99.5%, nausea 77.7%, fatigue 49.5%, alopecia 48.4%, vomiting 45.0%, constipation 35.9%, neutropenia 34.8%, decreased appetite 31%, anemia 29.9%, diarrhea 29.3% leukopenia 21.2%, thrombocytopenia 21.2% headache 19.6%, cuff 90%, abdominal pain 16.8%, lymphopenia 14.1%, interstitial lung disease 13.6%, QT prolongation 4.9%, infusion related reaction 2.2%, decreased EF 1.6%	Any adverse event 52.2%, neutropenia 20.7%, anemia 8.7% nausea 7.6%, fatigue 6.0%, vomiting 4.3%, leukopenia 6.0%, thrombocytopenia 3.8%, lymphopenia 6.0%, decreased appetite 1.6%, interstitial lung disease 2.7%, QT prolongation 1.1%, decreased LVEF 0.5%, alopecia 0.5%	Any event 15.2%, dose reduction 23.4%, pneumonitis 5.9%, interstitial lung disease 2.7%	0.21%	[24]
	DESTINY-Breast03 (n = 261)	Any event 99.5%, nausea 73%, fatigue 47.7%, alopecia 37.7%, vomiting 34%, neutropenia 33.2%, anemia 33.2%, increased LFTs 23.5%, thrombocytopenia 23.7%, leukopenia 23.2%, diarrhea 22.4%, constipation 21.3%, decreased appetite 20.6%, pneumonitis 12.1%	Any event 52.6%; neutropenia 13.7%, pneumonitis anemia 8.1%, thrombocytopenia 5.1%, fatigue 7.5%, leukopenia 6.5%, decreased appetite 2.4%, increased LFTs 3.2%, nausea 4.6%, vomiting 1.3%, pneumonitis 2.1%, diarrhea 1.1%	Not reported	0.8%	[7]
	DESTINY-Lung01 (n = 91)	Any adverse event 97%, nausea 73%, fatigue 53%, alopecia 46%, vomiting 40%, neutropenia 35%, anemia 33%, diarrhea 33%, decreased appetite 30%, interstitial lung disease 26%, leukopenia 23%, constipation 20%.	Any adverse event 46%, neutropenia 18%, any adverse event 46%, anemia 10%, nausea 9%, fatigue 7%, leukopenia 4%, vomiting 3%, diarrhea 3%	Dose reduction 34%, dose interruption 32%, %, treatment discontinuation 25%, pneumonitis 13%, interstitial lung disease 5%	2.91%	[5]
Enfortumab vedotin (Padcev) or EV	EV-301 (n = 296)	Any adverse event 93.9%, skin reactions 47%, peripheral neuropathy 46.3%, alopecia 45.3%, peripheral sensory neuropathy 33.8%, pruritus 32.1%, fatigue 31.1%, decreased appetite 30.7%, diarrhea 24.3% dysgeusia 24.3%, nausea 22.6%, anemia 11.5% decrease neutrophil count 10.1%, dry eye 15.9%, infusion related reactions 8.8%neutropenia 6.8%, hyperglycemia 6.4%, decreased leukocyte count 5.4%, febrile neutropenia 0.7%	Any adverse event 51.4%, maculopapular rash 7.4%, fatigue 6.4%, decreased neutrophil count 6.1%, neutropenia 4.7%, diarrhea 3.4%, peripheral sensory neuropathy 3.0%, pruritus 1.4%, decreased white cell count 1.4%, anemia 2.7%, decreased appetite 3%, nausea 1%, febrile neutropenia 0.7%	Dose interruption 51%, dose reduction 32.4%, peripheral sensory neuropathy 15.5%, fatigue 5.4%, decreased neutrophil count 5.1%, rash 3.4%, peripheral neuropathy 3%, anemia 2.7%, drug eruption 2.4% neutropenia 2%, diarrhea 2%, asthenia 2% increased ALT 2%	0.3%	[23]
Sacistuzuzmab Govitecan (SG)	ASCENT trial (n = 235)	Any adverse event 98%, neutropenia 63%, diarrhea 59%, nausea 57%, fatigue 45%, alopecia 46%, anemia 34%, vomiting 29%, nervous system disorders 25%, decreased appetite 20%, constipation 17%, leukopenia 16%, abdominal pain 11%, asthenia 12%, thrombocytopenia 5%, febrile neutropenia 6%	Any adverse event 64%, neutropenia 51%, leukopenia 10%, thrombocytopenia 2%, febrile neutropenia 6%, diarrhea 10%, nausea 3%, vomiting 3%, abdominal pain 1%, fatigue 3%, asthenia 1%	Dose reductions 22%, discontinuation 5%,	1.27%	[25]
Tisotumab vedotin (Tivdak) or TV	innovaTV 204 (n = 101)	Any adverse event 92%, ocular treatment-related events 53% (conjunctivitis 26%, dry eye 23%, keratitis 11%), bleeding 39% (epistaxis 20%, vaginal hemorrhage 7%, hematuria 3%), peripheral neuropathy 33%, alopecia 38%, nausea 27%, fatigue 26%, myalgia 15%, anemia 13%, asthenia 12%, arthralgia 2%, decreased appetite 11%, pruritus 10%, constipation 9%	Any adverse event 28%, neutropenia 3%, fatigue 2%, ulcerative keratitis 2%, peripheral neuropathy 2%, bleeding 2%	Dose interruption 24%, dose reduction 22%, treatment discontinuation 12%, serious adverse events 31%, peripheral neuropathy 2%, pyrexia 2%	0.9%	[27]
Mirvetuximab soravtansine	FDA approval notice and phase I safety study (n = 64)	Diarrhea 43.5%, blurry vision 41.3%, nausea 37%, fatigue 30%, neuropathy 28.3%, keratopathy 26%, AST increased 23.9%, ALT increased 15.2%, vomiting 21.7%, dry 30%, anemia 30%, headache 10.9%, hypokalemia 10.9%, hypomagnesemia 10.9%	Any adverse event 26%, fatigue 4%, hypotension 4%, febrile neutropenia and septic shock 1/64,	Serious adverse events 22%	0	[66]

## Supplementary Materials

The following supporting information can be downloaded at: https://www.mdpi.com/article/10.3390/pharmaceutics15082160/s1, Figure S1: References for pharmacokinetic data in Figure 3. References [106,107,108,109,110,111,112,113,114,115] are cited in Supplementary Materials.
