# Supplementary material for: Antibody-Drug Conjugates in Solid Tumor Oncology: An Effectiveness Payday with a Targeted Payload
Source: Pharmaceutics. 2023 Aug 19;15(8):2160. doi: 10.3390/pharmaceutics15082160 (PMC10459723; doi:10.3390/pharmaceutics15082160)
Supplement: Supplementary file 1 [file pharmaceutics-15-02160-s001.zip › pharmaceutics-2487361-supplementary.pdf]

Supplemental Figure S1. References for pharmacokinetic data in Fig 3

Drug	IC50 lower (nM)	IC50 upper (nM)	Reference
Paclitaxel	2.5	7.5	[1, 2]
Calicheamicin	0.1	3.58	[3]
MMAE	1	4	[4, 5]
Duocarmycin	0.07	0.22	[6]
5-fluorouracil	3000	39000	[7]
Doxorubicin	631	2670	[1]
Irinotecan	5000	15800	[8]
Pembrolizumab	0.1	1	[9, 10]

Uncategorized References

1. Yang, W., et al., *Genomics of Drug Sensitivity in Cancer (GDSC): a resource for therapeutic biomarker discovery in cancer cells.* Nucleic Acids Res, 2013. **41**(Database issue): p. D955-61.
2. Liebmann, J.E., et al., *Cytotoxic studies of paclitaxel (Taxol) in human tumour cell lines.* Br J Cancer, 1993. **68**(6): p. 1104-9.
3. de Vries, J.F., et al., *The novel calicheamicin-conjugated CD22 antibody inotuzumab ozogamicin (CMC-544) effectively kills primary pediatric acute lymphoblastic leukemia cells.* Leukemia, 2012. **26**(2): p. 255-64.
4. Burns, K.E., M.K. Robinson, and D. Thevenin, *Inhibition of cancer cell proliferation and breast tumor targeting of pHLIP-monomethyl auristatin E conjugates.* Mol Pharm, 2015. **12**(4): p. 1250-8.
5. Doronina, S.O., et al., *Development of potent monoclonal antibody auristatin conjugates for cancer therapy.* Nat Biotechnol, 2003. **21**(7): p. 778-84.
6. Elgersma, R.C., et al., *Design, Synthesis, and Evaluation of Linker-Duocarmycin Payloads: Toward Selection of HER2-Targeting Antibody-Drug Conjugate SYD985.* Mol Pharm, 2015. **12**(6): p. 1813-35.
7. Ando, T., et al., *Relationship between expression of 5-fluorouracil metabolic enzymes and 5-fluorouracil sensitivity in esophageal carcinoma cell lines.* Dis Esophagus, 2008. **21**(1): p. 15-20.
8. Cui, Z.J., et al., *Systems Pharmacology-Based Precision Therapy and Drug Combination Discovery for Breast Cancer.* Cancers (Basel), 2021. **13**(14).
9. Chatterjee, M., et al., *Systematic evaluation of pembrolizumab dosing in patients with advanced non-small-cell lung cancer.* Ann Oncol, 2016. **27**(7): p. 1291-8.
10. Ho, W.J. and R. Mehra, *Pembrolizumab for the treatment of head and neck squamous cell cancer.* Expert Opin Biol Ther, 2019. **19**(9): p. 879-885.
